# Generation of Transgenic Mice that Conditionally Overexpress Tenascin-C

**DOI:** 10.3389/fimmu.2021.620541

**Published:** 2021-03-08

**Authors:** Saori Yonebayashi, Kazuko Tajiri, Mari Hara, Hiromitsu Saito, Noboru Suzuki, Satoshi Sakai, Taizo Kimura, Akira Sato, Akiyo Sekimoto, Satoshi Fujita, Ryuji Okamoto, Robert J. Schwartz, Toshimichi Yoshida, Kyoko Imanaka-Yoshida

**Affiliations:** ^1^Department of Cardiology, Faculty of Medicine, University of Tsukuba, Tsukuba, Japan; ^2^Department of Pathology and Matrix Biology, Graduate School of Medicine, Mie University, Tsu, Japan; ^3^Research Center for Matrix Biology, Mie University, Tsu, Japan; ^4^Department of Animal Genomics, Functional Genomics Institute, Mie University Life Science Research Center, Tsu, Japan; ^5^Department of Cardiology, Graduate School of Medicine, Mie University, Tsu, Japan; ^6^Department of Biology and Biochemistry, University of Houston, Houston, TX, United States

**Keywords:** matricellular protein, Cre-Lox, Atp8a2, heart development, myocardial inafrction, Nkx2.5, alpha myosin heavy chain

## Abstract

Tenascin-C (TNC) is an extracellular matrix glycoprotein that is expressed during embryogenesis. It is not expressed in normal adults, but is up-regulated under pathological conditions. Although TNC knockout mice do not show a distinct phenotype, analyses of disease models using TNC knockout mice combined with *in vitro* experiments revealed the diverse functions of TNC. Since high TNC levels often predict a poor prognosis in various clinical settings, we developed a transgenic mouse that overexpresses TNC through Cre recombinase-mediated activation. Genomic walking showed that the transgene was integrated into and truncated the *Atp8a2* gene. While homozygous transgenic mice showed a severe neurological phenotype, heterozygous mice were viable, fertile, and did not exhibit any distinct abnormalities. Breeding hemizygous mice with Nkx2.5 promoter-Cre or α-myosin heavy chain promoter Cre mice induced the heart-specific overexpression of TNC in embryos and adults. TNC-overexpressing mouse hearts did not have distinct histological or functional abnormalities. However, the expression of proinflammatory cytokines/chemokines was significantly up-regulated and mortality rates during the acute stage after myocardial infarction were significantly higher than those of the controls. Our novel transgenic mouse may be applied to investigations on the role of TNC overexpression *in vivo* in various tissue/organ pathologies using different Cre donors.

## Introduction

Tenascin-C (TNC) is a large extracellular matrix (ECM) glycoprotein and an original member of ‘matricellular proteins’ together with thrombosondin-1 (TSP1) and SPARC (secreted protein acidic and rich in cysteine; osteonectin) (1). Matricellular proteins are a growing family of unique ECM proteins that do not directly contribute to the formation of structural elements and are strongly up-regulated and modulate cellular functions during tissue remodeling under normal and pathological conditions (2–5). As a typical matricellular protein, TNC is transiently expressed at several steps during embryogenesis, is weakly expressed in normal adults, and is up-regulated under pathological conditions. The essential biological roles of TNC, particularly in morphogenesis, have been suggested based on its limited spatiotemporal expression pattern. Similar to the majority of matricellular proteins, global TNC knockout mice do not show a distinct phenotype (2, 6), which facilitated the misinterpretation in the 90s that it does not have any significant role. However, with careful analyses of TNC-deficient mice, some behavioral aspects subsequently emerged and also correlated with the findings of electrophysiological and morphometric analyses (7–9). Furthermore, disease models using TNC knockout mice revealed its important roles in tissue repair after injury, inflammation and cancer invasion (10–16). Combined with the findings of *in vitro* experiments, the administration of TNC purified from culture supernatants or recombinant TNC and its fragments to animal models (17–22) or the transfection of the TNC gene into cells (16, 23) revealed multiple roles for TNC as well as its receptors and signaling cascades. Accumulating evidence suggests that TNC has diverse functions and may exert harmful and beneficial effects on tissue repair in a context-dependent manner.

In the heart, proinflammatory and profibrotic-induced dysfunctions mediated by excess TNC have attracted attention because high serum levels of TNC have been shown to predict a poor prognosis in various clinical settings, such as after myocardial infarction (MI) and dilated cardiomyopathy [reviewed in (24)].

The expression of TNC in the heart is strictly limited to specific stages and sites during early embryonic development and also to restricted lesions during the acute stage of tissue repair in adult hearts, which suggest that the rapid elimination of TNC is crucial for maintaining homeostasis in the heart.

To simulate an *in vivo* environment with excess TNC, we developed a transgenic mouse that overexpresses TNC regulated by cre-lox conditional activation. The transgene was constructed using CAG promoter-driven mouse *Tnc* cDNA, in which the loxP-tagged stuffer gene was intercalated. By breeding transgenic mice with two types of Cre mice, we successfully induced the heart-specific overexpression of TNC in both cases.

## Methods

### Generation of the Mutant Strain

We utilized the *Cre/loxP* system to generate transgenic mice that conditionally overexpress tenascin-C. The insertion of 11-kb mouse tenascin-C cDNA into the PmeI site of pCAG-XstopX-polyA (25). was performed as described previously (25). Founders were made using a pronuclear injection into C57BL/6J zygotes. Mice heterozygous for the transgene were backcrossed to C57BL/6N for at least ten generations to produce the transgenic mouse strain, namely, C57BL/6N-*Tg (CAG-flox-Tnc)IYM1*.

### Genomic Walking for Chromosomal Mapping of the Transgene

The genomic DNA of the transgenic mouse was extracted and purified from the tail using the High Pure PCR Template Preparation Kit (Roche Life Science). The genomic sequences that flanked the transgenes were elucidated by genomic walking using the Universal Genome Walker™ kit (BD Bioscience Clontech, Palo Alto, CA) with a slight modification. Adaptor-ligated genomic DNA libraries of the transgenic lines were constructed with tail DNA digested with six restriction enzymes: Dral, ScaI, PvuIl, EcoRV, SmaI, and StuI (Takara Bio Inc., Tokyo, Japan). Primary PCR amplification was performed with an outer transgene-specific primer (5′-CCA GGC GGG CCA TTT ACC GTA AGT TAT-3′) for the CAG promoter and the outer adaptor primer provided in the kit. All PCR amplifications were performed using the HotStarTaq Master Mix (Qiagen, Tokyo, Japan) in a thermal cycler (PC806, Astec). After a 15-min incubation at 95°C for activation, primary PCR amplification was performed by 30 cycles at 94°C for 2 s and 68°C for 5 min. The primary PCR mixture diluted 100 times was used as the template for nested PCR amplification with a nested transgene-specific primer (5′-GGC GGG CCA TTT ACC GTA AGT TAT GT-3′) and the nested adaptor primer provided in the kit. Nested PCR amplification was performed at 95°C for 15 min, followed by 30 cycles at 94°C for 2 s and 68°C for 5 min. The nested PCR product was separated by electrophoresis on a 2% agarose gel in TAE buffer and visualized with ethidium bromide staining. The PCR product containing the flanking sequence was found in the Sca I library and extracted from gels using the MinElute Gel Extraction kit (Qiagen). Purified DNA was cloned using the TOPO PCR cloning kit (ThermoFisher Scientific). The nucleotide sequence of cloned DNA was elucidated by a commercial laboratory (BioMatrix Research, Nagareyama, Chiba, Japan). The transgene insertion sites in chromosomes were identified using a BLAST search via the Internet (https://blast.ncbi.nlm.nih.gov/Blast.cgi). Genomic walking analyses that proceeded backwards from the tails of the transgene were performed with two primers (5′-ACA TGG TCA TTC TCC GAG CCA GCT GT-3′ and 5′-TGG GCT GCT TCC TAA TGC AGG AGT-3′) in combination with the adapter primers provided in the kit. The PCR product containing the flanking sequence was detected in the PvuII library.

To detect the truncated forms of the gene products, the 3′ rapid amplification of cDNA ends (3′ RACE) method was performed using the 3′-Full RACE Core Set (Takara Bio Inc., Shiga, Japan) according to the manufacturer's instructions.

### Genotyping of Transgenic Mice

**Figure 1:**
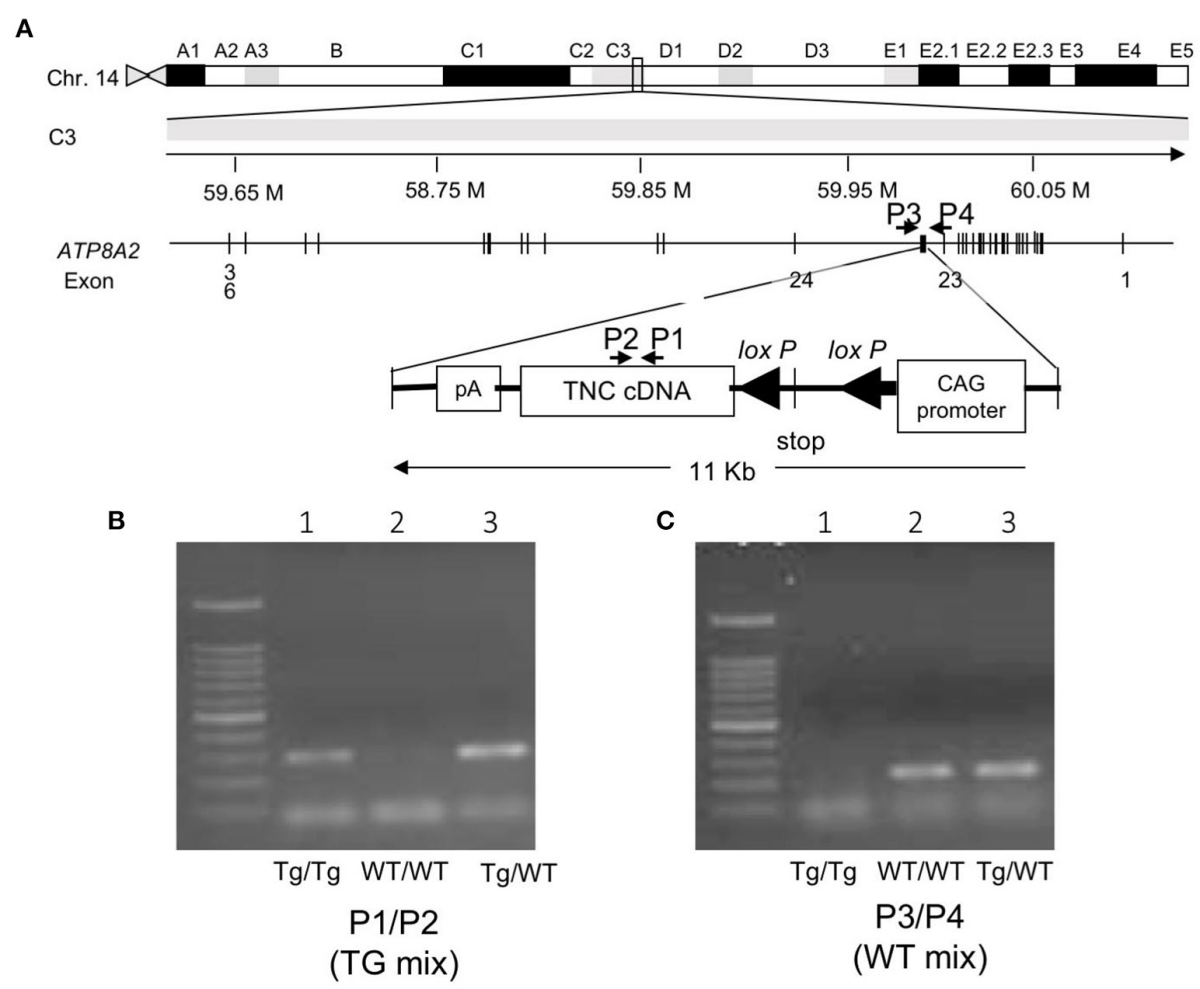
**(A)** Mapping the transgene. The transgene is integrated into an intron between exons 23 and 24 of the *Atp8a2* gene, encoding the murine phosphatidylserine translocase (flippase), on chromosome 14. **(B)** Gel image of the PCR product of the genotyping of mice with the primer set P1/P2 (TG mix). Tg/Tg and Tg/WT show the amplified transgene of *Tnc* as 298 bp. **(C)** Gel image of the PCR product of the genotyping of mice with the primer set P3/P4(WT mix). Tg/ WT and WT/WT show amplified wild-type alleles as 228 bp. Tg/Tg, homozygous; Tg/WT, hemizygous; WT/WT, wild type.

Two primers were designed to detect the transgene of TNC cDNA (Figure 1A, P1: 5′-AGG GTT GCC ACC TAT TTG C-3′ and P2: 5'-GCA TCC AGG CGG GTT GTG GTT AC-3'). Primers were also employed to detect the wild allele (Figure 1A, P3: 5′-AGG AGG GTC ACC AAC TGG CCT G-3′, P4: 5' -GGA CAG TGC TCT CAC TTG CCT GG-3'). The genotyping of the transgenic mouse C57BL/6N-*Tg (CAG-flox-Tnc)IYM1* (Tg) was conducted using PCR analyses of tail DNA under step-down PCR conditions for the primer pair P1 and P2 (TG mix): 95°C for 15 min, 2 cycles of 95°C 20 s/65°C 20 s/72°C 1 min, 2 cycles of 95°C 20 s/62°C 20 s/72°C 1 min, 2 cycles of 95°C 20 s/59°C 20 s/72°C 1 min, and 2 cycles of 95°C 20 s/56°C 20 s/72°C 1 min, followed by 30 cycles of 95°C 20 s/55°C 20 s/72°C 1 min. The PCR conditions used for the pair P3 and P4(WT mix) were as follows: 95°C for 15 min, 30 cycles of 95°C 20 s/62°C for 1 min, and 72°C for 7 min.

### Cre Mouse

*Nkx2.5* ^*Cre*^mice (Nkx2.5-Cre mice) were described previously (26). *Tg(Myh6-cre)2182 Md/J* mice (αMHC-Cre mice) (27) were kindly gifted from Professor M. D. Schneider.

The genotypes of mice were confirmed by a PCR analysis using the following primers: *Nkx2-5* primers (forward: 5′- CGGCATAGGACCAGAGTGATA-3′, reverse: 5′- TCCCTGAACATGTCCATCAGGTTC-3′); αMyHC Cre primer (forward: 5′-ATGACAGACAGATCCCTCCTATCTCC-3′, 5′-reverse:-CTCATCACTCGTTGCATCATCGAC-3′).

All animal experiments were approved by the Institutional Animal Experiment Committee of Mie University and the University of Tsukuba and conformed to the NIH Guide for the Care and Use of Laboratory Animals.

### Western Blot Analysis

Heart, lung, kidney, and skeletal muscle tissues were homogenized in RIPA buffer on ice using MagNA Lyser Green Beads (Roche Diagnostics, Indianapolis, IN, USA). The homogenate was centrifuged at 13,000 rpm at 4°C for 30 min. The protein concentration of supernatants was measured using the BCA Protein Assay Kit (Pierce, Rockford, IL, USA). Samples were separated by sodium dodecyl sulfate-polyacrylamide gel electrophoresis on 7–15% polyacrylamide gradient gels and transferred onto a polyvinylidene difluoride membrane. The membrane was blocked with 3% skim milk in tris-buffered saline (TBS) containing 0.1% Tween 20 (TBST), incubated with primary antibodies (1:10,000) in TBST at 4°C overnight, and then reacted with a horseradish peroxidase-conjugated goat anti-rabbit antibody (#7074, Cell Signaling Technology, Boston, MA, USA) in TBST at 25°C for 60 min. The following primary antibodies were used: TNC (#10337 clone 4F10TT, IBL, Japan), Cre (#69050-3, Novagen, San Diego, CA, USA), and tubulin (#2148, Cell Signaling Technology, Boston, MA, USA). Blots were visualized with a chemiluminescent reagent (ImmunoStar, Wako, Osaka, Japan) and the CCD camera system (Light-Capture II, Atto Co., Tokyo, Japan).

### Whole-Mount Immunostaining and Histological Examinations

Whole-mount immunostaining of mouse embryos was performed as previously described (28, 29). A polyclonal rabbit anti-TNC antibody (30) or monoclonal rat anti-PECAM antibody (clone MEC 13.3 BD Pharmingen, San Jose, CA, USA) was used at a dilution of 1:500. Regarding histological sections, adult mouse hearts were fixed in 4% paraformaldehyde in phosphate-buffered saline (PBS) and embedded in paraffin wax. Three-micrometer-thick sections were cut, and hematoxylin and eosin (H&E) staining or picrosirius red staining was performed. Immunostaining for TNC was conducted as previously described (31). Briefly, sections were treated with 0.4% pepsin (1:60,000; Sigma Chemical Corp., St. Louis, MO, USA) in 0.01 N HCl at 37°C for 10 min for antigen retrieval.

Evaluations of interstitial collagen fibers in picrosirius red-stained sections were performed as previously described (32). Mean cardiomyocyte diameters were also measured in H&E-stained sections by tracing 100 myocytes for each heart.

### RNA Extraction and Quantitative Reverse-Transcription Polymerase Chain Reaction

All hearts removed for qRT-PCR were snap-frozen and stored at −80°C. To prepare total RNA, tissue was homogenized using a bead kit (MagNA Lyser Green Beads; Roche Diagnostics, Indianapolis, IN, USA) according to the manufacturer's instructions. Total RNA samples from heart tissue and cultured cells were prepared using an RNeasy Mini Kit (Qiagen, Hilden, Germany). Complementary DNA was synthesized from 1 μg total RNA with a High Capacity cDNA Reverse Transcription kit (Applied Biosystems, Waltham, MA). The qRT-PCR analysis was performed using the LightCycler® 480 system (Roche Applied Science, Penzberg, Germany) with a Universal Probe Library (Roche Applied Science, Penzberg, Germany). Hypoxanthine-guanine phosphoribosyltransferase (*Hprt*) RNA was used as an internal control. Gene expression values were calculated using the 2^−ΔCt^ method.

### Echocardiography

Transthoracic echocardiography was performed with a Vevo 2100 instrument (Fujifilm Visual Sonics, Tokyo, Japan) equipped with an MS-400 imaging transducer. Isoflurane induction was performed in an induction box with 3% isoflurane in pure medical oxygen. After the righting reflex of mice waned, they were fixed in the supine position on a heating pad to maintain normothermia, followed by the placement of electrocardiographic limb electrodes. Anesthesia was maintained using 1% isoflurane.

### Induction of Myocardial Infarction

Myocardial infarction (MI) was induced using the classical MI method (33). Briefly, 10-week-old male mice weighing at least 25 g were anesthetized by an intraperitoneal injection of ketamine/xylazine (100–120 mg/kg body weight for ketamine and 7–8 mg/kg body weight for xylazine), intubated, and connected to a ventilator (Mouse Ventilator Minivent Type 845; Harvard Apparatus, Holliston, MA). The chest cavity was opened via left thoracotomy to expose the heart, which allowed the left anterior descending coronary artery to be visualized by the unaided eye or with a magnifying glass, and it was then permanently ligated with a 7-0 nylon suture at the site of its emergence from the left atrium. Complete occlusion of the vessel was confirmed by the presence of myocardial blanching in the perfusion bed. Mice that died during recovery from anesthesia were excluded from the analysis.

### Enzyme-Linked Immunosorbent Assay (ELISA)

Heart tissues were homogenized with RPMI-1640 containing 2.5% FBS using MagNA Lyser Green Beads (Roche Diagnostics, Indianapolis, IN, USA). Supernatants were collected after centrifugation and stored at −80°C. TN-C concentrations in the supernatants were measured using the Tenascin-C Large (FNIII-B) Assay kit (IBL, Takasaki, Japan).

### Survival Analysis

In the survival analysis after MI, we used littermates by mating C57BL/6N-*Tg (CAG-flox-Tnc)IYM1*^*tg*/+^(Tg) with αMHC-Cre mice. Sixty-five MI mice were used in the survival analysis (-/-, *n* = 19; -/Cre, *n* = 14; Tg/-, *n* = 18; Tg/Cre, *n* = 14). After 14 days, all surviving mice were euthanized by a lethal intraperitoneal injection of sodium pentobarbital (200 mg/kg) or CO_2_ inhalation.

### Statistical Analysis

All data are expressed as the mean ± standard error of the mean (SEM). Normality was verified with the Shapiro-Wilk test. A one-way analysis of variance (ANOVA) with Tukey's *post hoc* test or a Kruskal-Wallis analysis with the *post-hoc* Steel-Dwass or Dunnett's test was used for multiple comparisons. Survival distributions were estimated by the Kaplan–Meier method and compared using the Log-rank test. *P* < 0.05 was considered to indicate significance. All statistical analyses were performed with JMP software (SAS Institute, Cary, NC).

## Results

### Mapping and Genotyping of Transgenic Mice

A BLAST search with the 5′-flanking sequence revealed the integration of the transgene into an intron between exons 23 and 24 of the *Atp8a2* gene, encoding murine phosphatidylserine translocase (flippase), on chromosome 14 (Figure 1A). Primer sets of P1/P2 (TG mix) (Figure 1B) and P3/P4 (WT mix) (Figure 1C) produced transgenic and wild-type alleles as 298- and 228-bp bands, respectively. PCR analyses with the designed primers revealed the clear genotyping of three mice as homozygous (lane 1) with a transgene band only, wild-type (lane 2) with a wild band only, and heterozygous (lane 3) with both bands (Figures 1B,C). Several truncated forms of the gene product of *Atp8a2* were detected by a 3′ RACE analysis (data not shown).

### Phenotypes of Transgenic Mice

Mice homozygous for Tg grew more slowly than their littermate controls and a prominent neurological deficit was observed. They developed body tremors, an abnormal gait, and epileptic form attacks. Despite supplementation of dry food with a soft moist diet that was placed on the cage floor to allow easy access, all homo-mutant mice died by 100 days. This phenotype is similar to that of a spontaneous mouse mutant with a mutation in the *Atp8a2* gene, which is known as wabbler-lethal (*wl*) (34). Heterozygous mice were viable, fertile, and did not show any distinct phenotype abnormalities. Therefore, we used only heterozygous mice as TG(+) in the present study.

### Nkx2.5-Cre-Driven TNC-Overexpressing Mice

**Figure 2:**
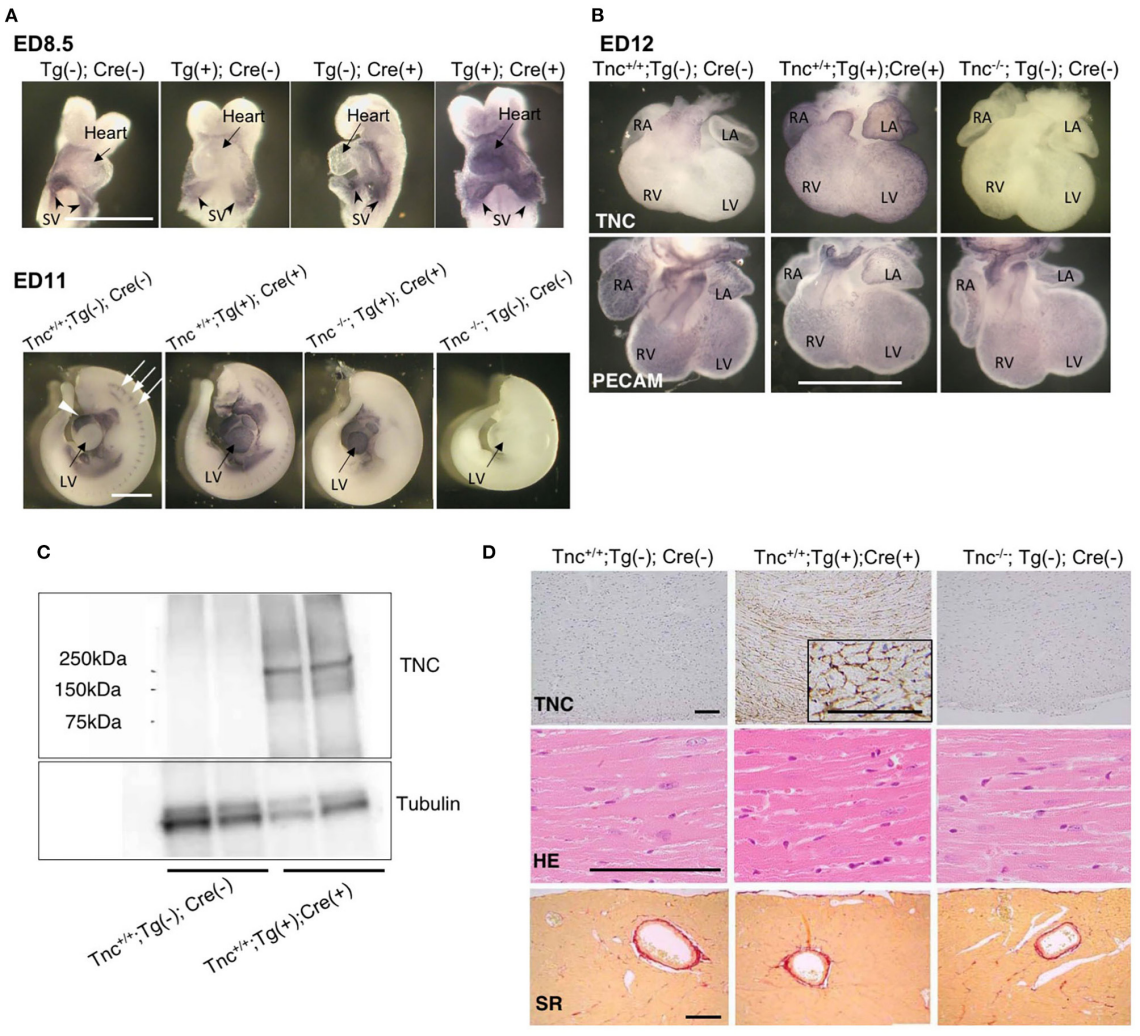
Heart-specific overexpression of TNC in mouse embryos induced by Nkx2.5-Cre. **(A)** Whole mount immunostaining for TNC in mouse embryos on ED8.5 and ED11. The intrinsic expression of TNC is observed in sinus venosus on ED8.5. The head and pericardium were removed from ED11 embryos. In the wild type (Tnc^+/+^;Tg(-); Cre(-)), the intrinsic expression of TNC was observed in the outflow tract of the heart (white arrow head) and at somites (white arrows). Scale bar = 1mm. **(B)** Whole-mount immunostaining of the mouse heart on ED12 for TNC and PECAM. Scale bar = 1 mm. **(C)** A western blot analysis of TNC protein expression in 64-week-old adult hearts. **(D)** Histological sections of the myocardium of 64-week-old mice stained with anti-TNC, H&E, and picrosirius red. Scale bar = 50 μm; SV, sinus venosus; LV, left ventricle; RV, right ventricle; LA, left atrium; RA, right atrium.

To examine the induction of TNC expression, we bred heterozygous C57BL/6N-*Tg (CAG-flox-Tnc)IYM1*^*tg*/+^ (Tg(+)) with heterozygous *Nkx2.5*^*Cre*/+^ (Cre(+)) driver mice. Nkx2.5-Cre drove efficient recombination in the embryonic heart. On embryonic day (ED) 8.5, the over-/misexpression of TNC was observed in the whole primitive heart tube of Tg(+); Cre(+) embryos, while the intrinsic expression of TNC was restricted to the sinus venosus (Figure 2A). TNC expression in Tg(+); Cre(-) and - Tg(-); Cre(+) mice was identical to that in the wild type Tg(-); Cre(-). Furthermore, by crossing TNC knockout mice with Tg(+); Cre(+) mice, we induced the expression of TNC only in the whole heart with the deletion of TNC in other tissues (Figure 2A, ED11). TNC-overexpressing embryonic mice developed grossly normal hearts. The development of coronary arteries also appeared to be normal in TNC over-/misexpressing embryos as well as in TNC-deficient mice (Figure 2B). No distinct difference in the coronary vasculature was found between heterozygous TG or Nkx2.5-Cre mice and the wild type (data not shown). Heart-specific TNC-overexpressing mice were viable at least until 64 weeks. TNC expression was detected in the adult heart at 64 weeks (Figure 2C) and positive immunostaining for TNC was observed in the intercellular spaces of the myocardium of the (Figure 2D). No significant differences were observed in histology, fibrosis, or cardiac function to compare with those of wild-type and TNC knockout mice even at 64 weeks old (Table 1).

**Table 1:** Histological and echocardiographic findings of the hearts of Nkx2.5C-Cre-induced TNC-overexpressing mice and TNC knockout mice (64 weeks old).

	**Tnc^**+/+**^;Tg(-); Cre(-) (Wild type)**	**Tnc^**+/+**^;Tg(+);Cre(+) (excess TNC)**	**Tnc^**−/−**^; Tg(-); Cre(-) (TNC defect)**	***P***
Male	(*n* = 7)	(*n* = 6)	(*n* = 3)	
BW (g)	35.4 ± 2.0	42.8 ± 1.9	38.5 ± 2.3	0.04
Heart weight (mg)	200.0 ± 8.6	216.8 ± 9.3	205.7 ± 13.1	0.44
Body Heart ratio (%)	5.7 ± 0.2	5.1 ± 0.2	5.1 ± 0.2	0.13
Cardiomyocyte size (mm)	65.5 ± 1.2	66.5 ± 1.3	64.8 ± 1.7	0.74
Collagen volume fraction(%)	16.2 ± 1.8	19.5 ± 1.9	15.7 ± 2.7	0.38
Echocardiography	(*n* = 3)	(*n* = 4)	(*n* = 4)	
PWTd (mm)	1.03 ± 0.08	1.20 ± 0.07	1.13 ± 0.07	0.34
LVDd (mm)	3.67 ± 0.31	3.83 ± 0.27	3.58 ± 0.27	0.81
EF (%)	31.5 ± 5.6	37.8 ± 4.8	44.3 ± 4.8	0.28
Female	(*n* = 6)	(*n* = 6)	(*n* = 4)	
BW (g)	30.7 ± 1.7	30.8 ± 1.7	29.0 ± 2.1	0.76
Heart weight (mg)	141.5 ± 10.9	172.2 ± 10.9	149.0 ± 13.3	0.15
Body Heart ratio (%)	4.6 ± 0.2	5.6 ± 0.2	5.1 ± 0.2	0.03
Cardiomyocyte size (mm)	65.4 ± 1.1	68.0 ± 1.1	65.0 ± 1.4	0.20
Collagen volume fraction(%)	13.4 ± 1.5	16.3 ± 1.5	12.0 ± 2.2	0.25
	(*n* = 3)	(*n* = 4)	(*n* = 4)	
Echocardiography				
PWTd (mm)	0.96 ± 0.11	1.03 ± 0.10	1.08 ± 0.10	0.77
LVDd (mm)	3.53 ± 022	3.40 ± 0.19	3.30 ± 0.19	0.74
EF (%)	38.3 ± 6.6	47.8 ± 5.8	38.8 ± 5.8	0.48

*Data are expressed as mean ± SEM. For multiple comparisons, one-way analysis of variance was used. BW, body weight; PWTd, posterior wall thickness in diastole; LVDd, left ventricular dimension in diastole; EF, ejection fraction*.

### αMHC-Cre-Driven TNC-Overexpressing Mice

**Figure 3:**
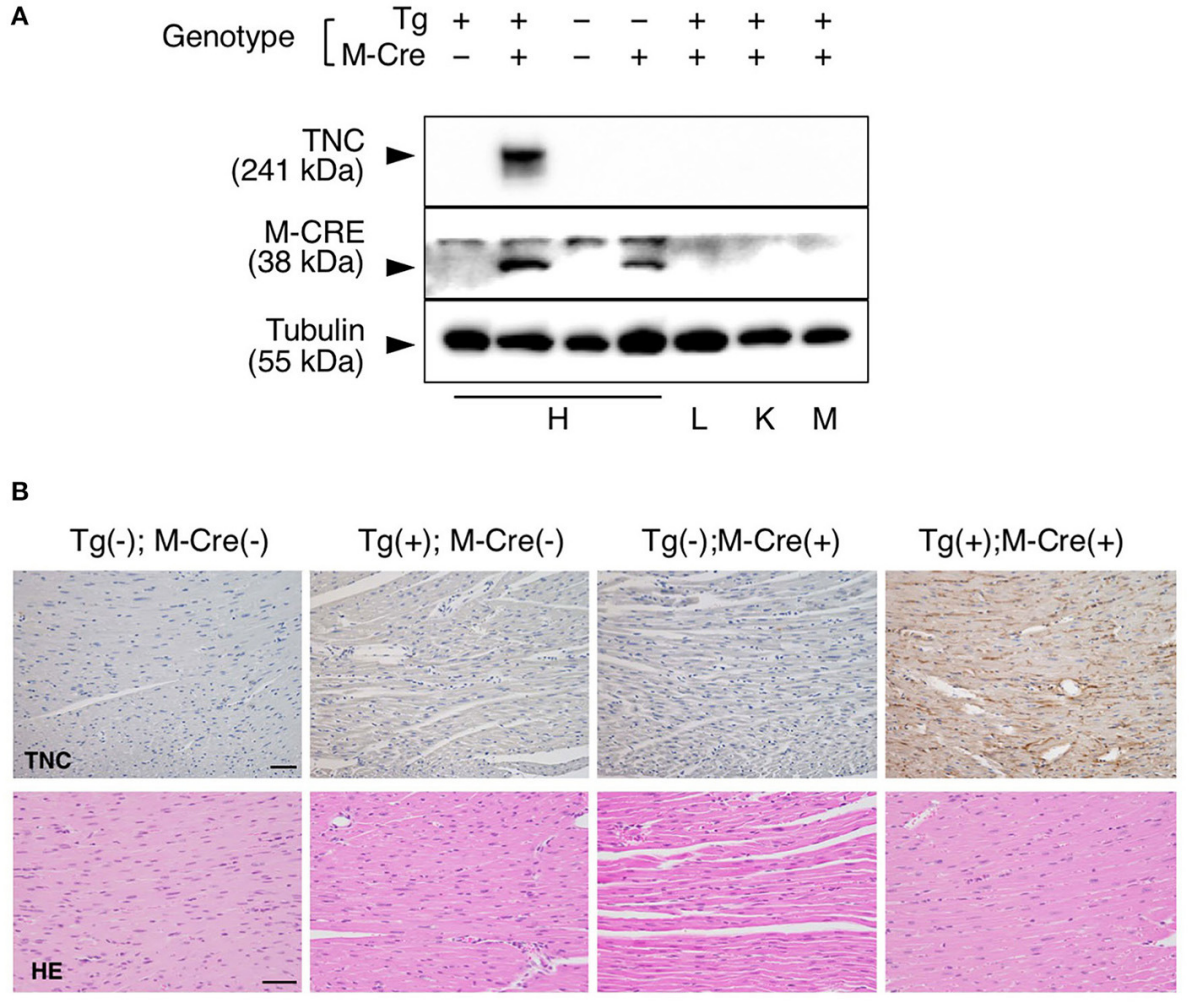
Heart-specific overexpression of TNC in adult mice induced by αMHC-Cre. **(A)** Western blot analysis of TNC protein expression in 10-week-old mice. TNC expression is induced only in heart of Tg(+); M-Cre(**+**) mouse but not in lung, kidney or skeletal muscle. H, heart; L, lung; K, kidney; and M, muscle. **(B)** Histological section of the myocardium stained with anti-TNC, H&E and picrosirius red. Scale bar = 50 μm.

**Figure 4:**
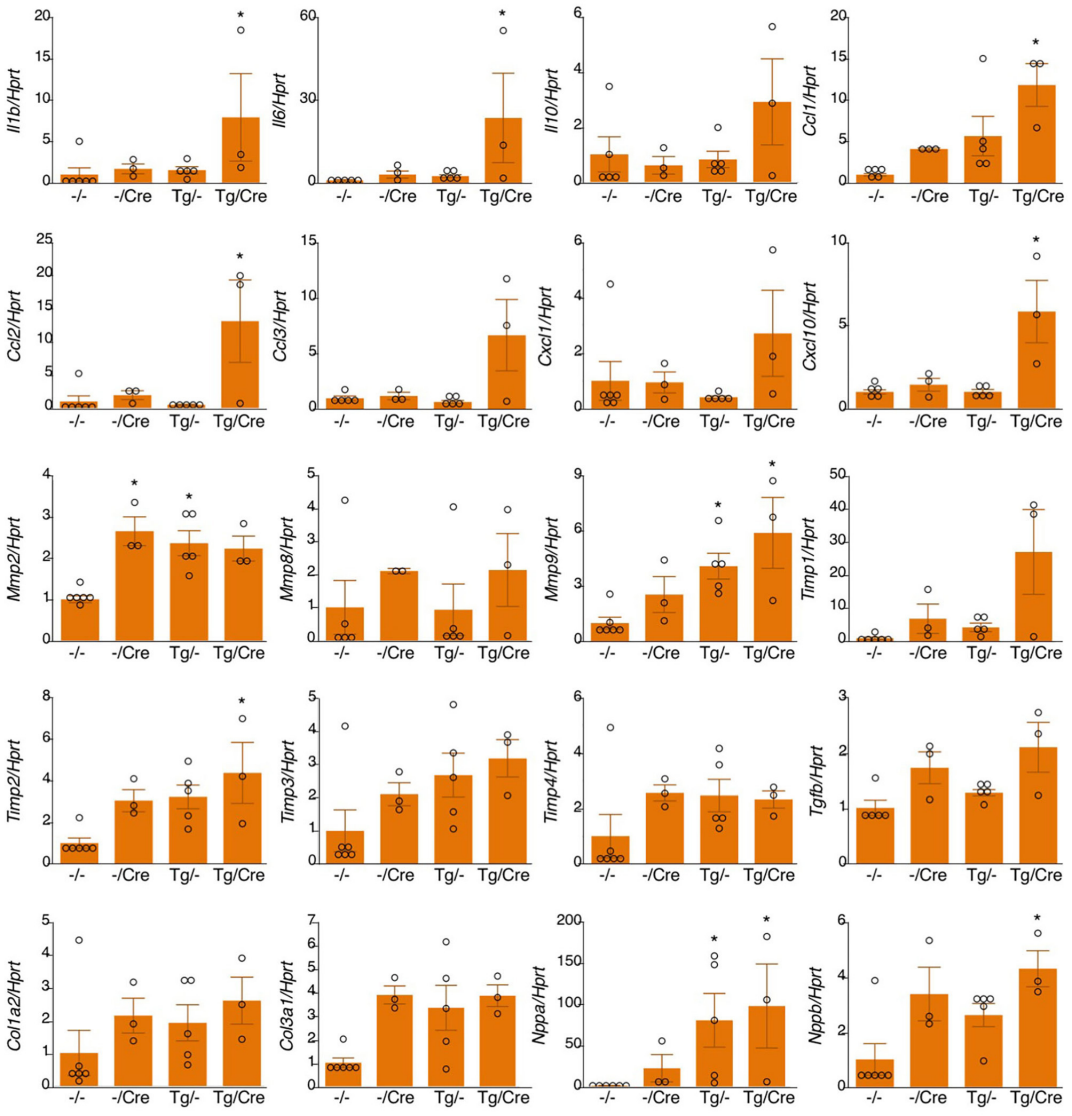
mRNA expression in αMHC-Cre-induced TNC-overexpressing hearts. Results are shown as mean ± SEM, *n* = 3–6, ^*^P < 0.05 vs. -/- by a Kruskal-Wallis analysis with the *post-hoc* Dunnett's test. -/-, Tg(-); M-Cre(-); Tg/-, Tg(+); M-Cre(-); -/Cre, Tg(-); M-Cre(+); Tg/Cre, Tg(+); M-Cre(**+**).

We bred heterozygous Tg mice with heterozygous *Tg(Myh6-cre)2182 Md/J*, a cardiomyocyte-specific α-myosin heavy chain promoter Cre mouse(M-Cre(+)). Tg(+);M-Cre(+)mice showed high mRNA and protein levels of myocardial TNC at 10 weeks old (Figure 3A). Tg(+);M-Cre(+) mice were born in Mendelian ratios, appeared healthy, and had normal cardiac function, size, and histology (Table 2). Although inflammatory cell infiltration was not observed in any mouse groups (Tg(+);M-Cre(+), Tg(+);M-Cre(-), Tg(-);M-Cre(+), Tg(-);M-Cre(-), Figure 3B). Tg(+);M-Cre(+) mice showed high mRNA expression levels of pro-inflammatory cytokines and chemokines (e.g., IL-1β, IL-6, CCL1, CCL2, and CXCL10) in the heart (Figure 4). Moreover, Tg(+);M-Cre(+) mice had high levels of tissue remodeling-related genes (MMP9 and TIMP2) and hypertrophy-related genes (ANP and BNP). Therefore, the hearts of mice with cardiomyocyte-specific TNC overexpression did not exhibit any morphological or functional abnormalities; however, the mRNA expression levels of pro-inflammatory cytokines, tissue remodeling-related genes, and hypertrophy-related genes were elevated.

**Table 2:** Echocardiographic parameters in αMHC-Cre-induced TNC-overexpressing mice (12 weeks old).

	**Tg(-); M-Cre(-) (Wild type)**	**Tg(-); M-Cre(+)**	**Tg(+); M-Cre(-)**	**Tg(+); M-Cre(+) (excess TNC)**	***P*-value**
Male	(*n* = 5)	(*n* = 7)	(*n* = 9)	(*n* = 7)	
BW (g)	25.0 ± 0.9	26.1 ± 0.8	26.1 ± 0.7	24.9 ± 0.8	0.54
HR (bpm)	459 ± 22	428 ± 19	394 ± 16	403 ± 19	0.12
LVDd (mm)	3.74 ± 0.16	3.74 ± 0.14	3.66 ± 0.12	3.86 ± 0.14	0.77
LVDs (mm)	2.47± 0.11	2.50 ± 0.09	2.37 ± 0.08	2.53 ± 0.09	0.60
IVSTd(mm)	0.77 ± 0.03	0.80 ± 0.03	0.71 ± 0.03	0.77 ± 0.03	0.17
PWTd(mm)	0.85 ± 0.05	0.86 ± 0.04	0.85 ± 0.04	0.82 ± 0.04	0.93
FS (%)	33.9 ± 2.88	32.8 ± 2.44	35.0 ± 2.15	34.1 ± 2.44	0.93
EF (%)	56.7 ± 2.23	63.4 ± 1.89	61.1 ± 1.67	58.6 ± 1.89	0.12
E (mm/s)	844 ± 69	898 ± 58	936 ± 51	935 ± 58	0.71
A (mm/s)	582 ± 55	549 ± 47	571 ± 41	649 ± 47	0.47
E/A	1.49 ± 0.10	1.68 ± 0.08	1.65 ± 0.07	1.45 ± 0.08	0.17

*Data are expressed as mean ± SEM. For multiple comparisons, one-way analysis of variance was used. BW, body weight; EF, ejection fraction; FS, fractional shortening; HR, heart rate; IVSTd, interventricular septal thickness in diastole; LVDd, left ventricular dimension in diastole; LVDs, left ventricular dimension in systole; PWTd, posterior wall thickness in diastole*.

**Figure 5:**
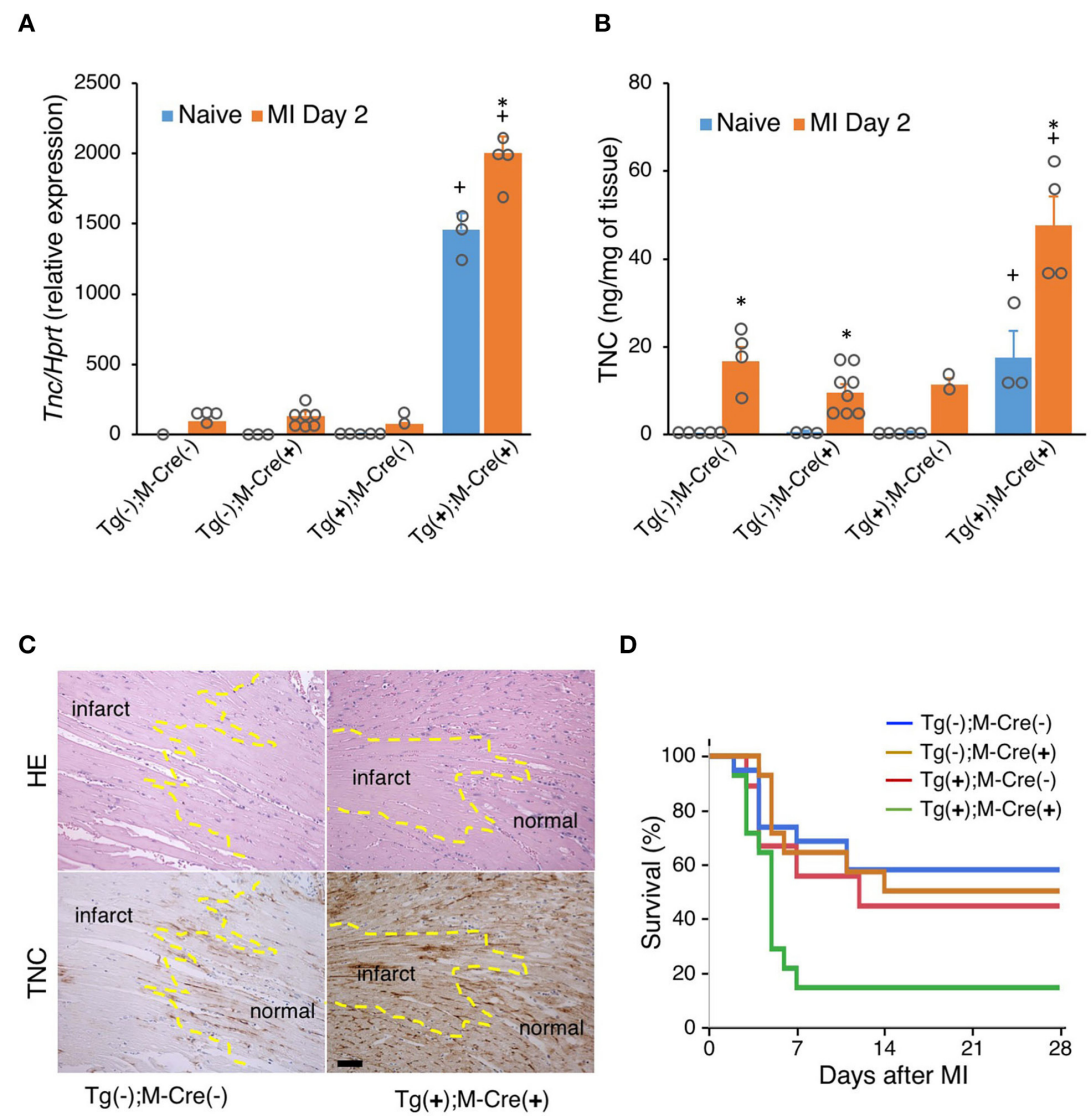
MI in mice with the heart-specific overexpression of TNC induced by αMHC-Cre. **(A)** TNC mRNA expression in naïve hearts and on day 2 after MI (*n* = 1–7). **(B)** TNC protein levels in naïve hearts and on day 2 after MI (*n* = 2–8). Results are shown as means ± SEM. ^+^*P* < 0.05 vs Tg(-); Cre(-), ^*^P < 0.05 vs Native **(C)** Immunostaining for TNC expression in the heart. Scale bar = 50 μm. **(D)** Kaplan–Meier survival analysis 28 days after MI. Blue line for -/- (*n* = 19), brown line for -/Cre (*n* = 14), red line for Tg/- (*n* = 18), and green line for Tg/Cre (*n* = 14).

We also investigated the effects of MI in this mouse. TNC expression levels were higher in all MI mice (Tg(+);M-Cre(+), Tg(+);M-Cre(-), Tg(-);M-Cre(+), Tg(-);M-Cre(-)) than in naïve mice; however, they were markedly higher in Tg(+);M-Cre(+) mice (Figures 5A,B). In wild-type MI mice, an immunohistochemical analysis of hearts on day 2 revealed that TNC was expressed at the borders between intact myocardial tissues and necrotic areas. In contrast, Tg(+);M-Cre(+) MI mice showed high TNC expression levels in both the infarct and normal areas of the heart (Figure 5C). Four-week survival rates were significantly lower in Tg/Cre mice than in other mice (Tg(+);M-Cre(+), 14.3% Tg(-);M-Cre(-), 57.9%; Tg(-);M-Cre(+), 50.0%; Tg(+);M-Cre(-), 44.4%, *P* = 0.014 by the Log-rank test, Figure 5D).

## Discussion

We generated a transgenic mouse that conditionally overexpresses TNC through Cre recombinase-mediated activation. By breeding heterozygous Tg mice with heterozygous Nkx2.5-Cre or αMyHC-Cre mice, we induced the heart-specific overexpression of TNC.

The *Nkx2-5* transcription factor is one of the earliest cardiogenic markers expressed in early heart mesoderm lineage progenitors and continues to be expressed in cardiomyocytes at later stages (35). The Nkx2.5-Cre mouse is often used to inactivate target genes in the early cardiac crescent on ED 7.5 (35–37).

We initially used Nkx2.5-Cre mice to examine the role of TNC during early heart development. TNC is normally expressed in precardiac mesodermal cells in the cardiac crescent; however, its expression is immediately down-regulated when mesodermal cells differentiate into cardiomyocytes, except in the outflow tract (28). We expected the prolonged expression of TNC in cardiomyocytes to perturbate heart morphogenesis. Although Nkx2.5-Cre drove TNC over-/misexpression in cardiomyocytes, the heart tube formed and underwent looping to produce a 4-chambered heart without any apparent abnormalities. We then focused on coronary vasculogenesis. In the normal mouse heart on ED 12, primitive coronary vascular networks are formed and cover the entire heart surface, except the TNC-positive outflow tract (24). With the shortening of the TNC-positive outflow tract, the vascular plexus gradually reaches the base of the aorta to form the proximal region of coronary arteries. This spatiotemporal relationship indicates that TNC may demarcate border zones and guide the developing vascular network. However, neither the complete deletion nor over-/mis-expression of TNC exerted apparent effects on the development of coronary vessels as shown in Figure 2. *Considering its pleiotropic function, excess TNC may be compensated for by other factors, particularly during development*, similar to the subtle phenotype of germinal KO mice.

The Nkx2.5-Cre-driven overexpression of TNC persisted in the adult mouse heart. The deposition of TNC in the extracellular spaces of the interstitium was immunohistochemically confirmed. The TNC-overexpressing heart did not show apparent changes in cardiac function or the histology of the myocardium, such as hypertrophy or fibrosis, between wild-type and TNC knockout mice, at least at 64 weeks old, as shown in Figure 3.

We used another Cre mouse to induce TNC in the adult heart. The α*MyHC* promoter is activated in cardiomyocytes on ED9.5 and genetic recombination by the α*MyHC*-cre construct in the heart is initiated by ED12.5 (38–40), which is slightly later than that by Nkx2.5-Cre. α*MyHC*-Cre is one of the most frequently used Cre donors (41) inducing heart-specific recombination in adults (36).

In the Tg(+);M-Cre(+) mouse, α*MyHC* specifically drove the overexpression of TNC in the heart, and TNC synthesized in cardiomyocytes was deposited in the intercellular spaces as well as in the Nkx2.5-Cre-driven TNC-overexpressing heart. Although no apparent histological change in the myocardium or inflammatory cell infiltration was detected in the naïve Tg(+);M-Cre(+) heart, a gene expression analysis revealed the significant up-regulation of proinflammatory cytokines/chemokines and MMPs as shown in Figures 3, 4. TNC is one of the proinflammatory molecules involved in myocardial tissue remodeling and activates fibroblasts (42–44) and macrophages (20, 33, 45–47) to up-regulate proinflammatory cytokines/chemokines and MMPs *in vitro*. Therefore, the present results suggest that TNC synthesized in cardiomyocytes by genetic engineering activated interstitial cells in a paracrine manner *in vivo*. Furthermore, TNC-overexpressing mice showed significantly higher mortality rates during the acute phase after MI that were associated with greater increases in TNC levels than in the controls, which also supports TNC potentially enhancing inflammatory responses after MI by making a positive feedback loop (24, 44). These findings indicate that TNC-overexpressing mice are a good model for investigating the biological role of TNC in the microenvironment of the pathological myocardium.

However, the results obtained in TNC-overexpressing mice need to be carefully interpreted. We used cardiomyocyte-specific Cre to induce the overexpression of TNC in the adult myocardium. As discussed earlier, the expression of TNC in cardiomyocytes is limited at the very early embryonic stage, and cardiomyocytes do not synthesize TNC but interstitial cells are its source in the adult pathological myocardium. The forced expression of TNC in cardiomyocytes may induce an abnormal cellular response. In the present study, although no significant histo/morphological changes were observed in cardiomyocytes, ANP and BNP, hypertrophy/stress markers of cardiomyocytes, were elevated in the TNC-overexpressing heart. Although TNC may up-regulate ANP and BNP expression by cardiomyocytes (48, 49), this may reflect aberrant stress in cardiomyocytes, such as endoplasmic reticulum (ER) stress, in addition to the extraneous autocrine stimulation by TNC. Furthermore, several pathological phenotypes in the hearts of the heterozygous Nkx2.5 knockout status (Cre knock-in mice) (50, 51) and the cardiotoxicity of prolonged Cre expression in α*MyHC-Cre mouse* mice (41) have been reported.

It should be also mentioned that homozygous Tg mice showed a severe phenotype, which was prominent in the nervous system, due to the truncation of the ATP8a2 gene by the insertion of the transgene. Although we did not detect any significant differences, at least in the hearts of heterozygous Tg mice, we need to considered the effects of truncated ATP8a2 particularly in the nervous system.

In conclusion, our novel Tg mice may be applied to investigations on the role of TNC overexpression under a number of tissue/organ pathologies using different Cre donors; however, appropriate control animals, such as mice carrying the cre transgene only, and heterozygous Tg mice are important for comparisons.

## Data Availability Statement

The original contributions presented in the study are included in the article/supplementary material, further inquiries can be directed to the corresponding author/s.

## Ethics Statement

The animal study was reviewed and approved by Institutional Animal Experiment Committee of Mice University and Institutional Animal Experiment Committee of Tsukuba University.

## Author Contributions

KI-Y and TY designed the study. KT, TY, and KI-Y wrote this manuscript. KI-Y, MH, and RS analyzed heart development. SF and RO analyzed cardiac function. HS and NS generated the transgenic mice. ASe and TY performed chromosomal mapping of the transgene. SY, KT, SS, TK, and ASa analyzed myocardial infarction model. All authors contributed to the article and approved the submitted version.

## Conflict of Interest

The authors declare that the research was conducted in the absence of any commercial or financial relationships that could be construed as a potential conflict of interest.
